# Embedding a surface acoustic wave sensor and venting into a metal additively manufactured injection mould tool for targeted temperature monitoring

**DOI:** 10.1007/s00170-023-12932-7

**Published:** 2024-01-25

**Authors:** Rokas Šakalys, Christopher O’Hara, Mandana Kariminejad, Albert Weinert, Mohammadreza Kadivar, Bruno Zluhan, Marion McAfee, Gerard McGranaghan, David Tormey, Ramesh Raghavendra

**Affiliations:** 1 I-Form, the SFI Research Centre for Advanced Manufacturing, University College Dublin, Room L0.13, CSCB Building, O’Brien Centre for Science (East), Belfield, Dublin 4, Ireland; 2 South Eastern Applied Materials Research Centre (SEAM), South East Technological University, Waterford, X91TX03 Ireland; 3 Centre for Precision Engineering Material and Manufacturing Research (PEM Research Centre), Atlantic Technological University, Ash Lane, Sligo, F91 YW50 Ireland

**Keywords:** Wireless sensor embedding, Print-in-place venting, Injection moulding, Additively manufactured injection mould tools, In-mould sensors

## Abstract

Injection moulding (IM) tools with embedded sensors can significantly improve the process efficiency and quality of the fabricated parts through real-time monitoring and control of key process parameters such as temperature, pressure and injection speed. However, traditional mould tool fabrication technologies do not enable the fabrication of complex internal geometries. Complex internal geometries are necessary for technical applications such as sensor embedding and conformal cooling which yield benefits for process control and improved cycle times. With traditional fabrication techniques, only simple bore-based sensor embedding or external sensor attachment is possible. Externally attached sensors may compromise the functionality of the injection mould tool, with limitations such as the acquired data not reflecting the processes inside the part. The design freedom of additive manufacturing (AM) enables the fabrication of complex internal geometries, making it an excellent candidate for fabricating injection mould tools with such internal geometries. Therefore, embedding sensors in a desired location for targeted monitoring of critical mould tool regions is easier to achieve with AM. This research paper focuses on embedding a wireless surface acoustic wave (SAW) temperature sensor into an injection mould tool that was additively manufactured from stainless steel 316L. The laser powder bed fusion (L-PBF) “stop-and-go” approach was applied to embed the wireless SAW sensor. After embedding, the sensor demonstrated full functionality by recording real-time temperature data, which can further enhance process control. In addition, the concept of novel print-in-place venting design, applying the same L-PBF stop-and-go approach, for vent embedding was successfully implemented, enabling the IM of defectless parts at faster injection rates, whereas cavities designed and tested without venting resulted in parts with burn marks.

## Introduction

Plastics demonstrate outstanding mechanical properties, low density and corrosion resistance. The characteristics mentioned above are the main reasons for the growth of the demand for plastic components and novel applications across industries like aerospace, automotive and healthcare. Therefore, not surprisingly, the polymer industry is the third largest manufacturing industry worldwide and is estimated to reach a market of US $750.1 billion in 2028 [1, 2]. Injection moulding (IM) is a manufacturing technology used to mass-produce polymer components for a range of products and applications. IM offers cost efficiency, automation potential and the ability to reliably produce components of high quality and dimensional accuracy with high repetition. Therefore, it is widely applied across many industries, accounting for approximately 80% of the total plastic industry merchandise worldwide [3–7].

Throughout the IM process, the polymer experiences changes in temperature. These thermal variations can cause variations in dimensional accuracy, weight and crystallinity of the fabricated parts [6] due to uneven cooling and residual stresses that form in the polymer. The cooling step is critical since it directly affects the part quality, where inappropriate cooling may result in defects such as shrinkage or warpage. The cooling stage can take up to 60% of the total IM cycle time. Therefore, it is beneficial to enhance the cooling cycle, thus achieving improved process efficiency, lower energy consumption and high quality of the parts [8]. Optimisation of the cooling cycle is a complicated task, which typically involves the design and optimised placement of conformal cooling channels specific to the mould cavity combined with adjusted process parameters [3, 9].

Alternatively, optimisation can be greatly enhanced by applying process control technologies such as artificial intelligence and machine learning approaches that use data from in-mould sensors, thus enabling IM via Industry 4.0 technologies. In such approaches, in-mould sensors play a critical role in the acquisition of vital process information; the closer to the polymer melt a sensor is placed, the richer the data. Typically getting sensors close to the polymer melt greatly increases the cost and complexity of the injection mould. There are a variety of in-mould sensors available depending on the parameters of interest; however, temperature and pressure sensors are the main two categories [10].

Numerous researchers have successfully utilised thermocouples for temperature monitoring during the IM process to obtain accurate thermal profiles and control the process [10–15]. The successful application of these sensors requires incorporating design features for the sensor body and wiring into the mould tool. However, as injection mould tools are typically designed and manufactured with relatively complicated geometry to reflect the complexity of the final component, the installation of additional sensors and wiring significantly increases the toolmaking costs and complexity. This is particularly true when multiple sensors are installed. Therefore, wireless sensors for IM process monitoring are a particularly attractive technology since they enable in-process data acquisition while avoiding the need to create additional mould modifications for wire routing in an already constrained area [16–19].

The power supply is critical for the functioning of a wireless sensor. While batteries are an option, battery replacement is a significant drawback in an industrial setting as it increases maintenance and cost [20]. Increasing the battery size to extend the sensor lifetime can likewise significantly increase the sensor size and the installation complexity in an already constricted space [18]. The high-temperature process environment can also be damaging to many types of batteries.

Wireless and passive surface acoustic wave (SAW) sensors are self-excited and do not require wiring or a power supply, making them an attractive alternative for measuring temperature, pressure and other parameters [21, 22]. SAW sensors can potentially eliminate many of the challenges mentioned above that are characteristic of other sensors. However, there is limited published information in the literature about SAW sensor applications in IM. Therefore, toolmaking and in-mould SAW sensor integration must be considered together to appreciate the challenges and benefits of using a SAW sensor in an IM process.

Internal geometries, such as pockets and channels, that are necessary for embedding the SAW sensor are problematic to achieve using traditional subtractive toolmaking methods such as CNC machining or even spark erosion [23, 24]. The layer-by-layer nature and design freedom offered by additive manufacturing (AM) enables the design and manufacturing of complex internal geometries. These can be designed without compromising the mechanical properties of the part. Therefore, in previous studies, AM was used to fabricate injection mould tools with conformal cooling channels and other internal geometries that would be impossible to fabricate using subtractive technologies [24, 25].

Successful sensor embedding into plastic 3D-printed parts was documented in previous studies [26]; however, metal AM processes typically attain high process temperatures, which can potentially damage sensitive electronic components. Therefore, an installation strategy that includes component protection when embedding wireless sensors into metal AM parts is necessary to overcome this limitation [27]. Several successful studies have demonstrated promising results while applying the so-called stop-and-go strategy for embedding electronic components into various additively manufactured parts. A stop-and-go process was applied in research work using electron beam melting to produce a smart metal component. Here a piezoelectric material was embedded into a metallic Ti–6Al–4 V additively manufactured component. The compression tests demonstrated the capability of the component with embedded piezoelectric material to sense external forces. This research indicated potential application of this smart component for energy harvesting [28].

Additional research using the stop-and-go approach, this time in a selective laser melting (SLM) AM process, successfully embedded a thermocouple sensor in an SS316L part and an acceleration measuring IC board in an Inconel 718 turbine blade. The study by Jung et al. demonstrated complete functionality of embedded sensors during laboratory testing with no signs of degradation from the embedding process [27]. Tomaz et al. successfully embedded a SAW temperature sensor into a stainless steel 316L component additively manufactured by laser powder bed fusion (L-PBF). The authors demonstrated the sensor functionality in lab tests to validate ability to receive the wireless signal of the temperature readings [19].

The aim of this research is to embed a wireless SAW temperature sensor into an injection mould tool using the stop-and-go technique used in previous works [19, 27]. The stop-and-go strategy for L-PBF was applied to embed the sensor and thermally protect the electronic components during the process. The sensor functionality was successfully validated in an IM process using an industrial 100-ton IM machine where the SAW sensor obtained real-time in-process data, as well the structural integrity of the mould was verified. This demonstrates the potential of applying this technology and embedding methods for enhanced IM process monitoring and control.

## Materials and methods

A production part, commercially manufactured by an industrial partner, was selected for this study as the component’s attributes are particularly sensitive to temperature variability. The standard core and cavity tooling designs were adapted from the commercial mould tool design. They underwent design modifications to make improvements that enabled AM of smart tooling by embedding a wireless SAW temperature sensor. The L-PBF stop-and-go method was used for the sensor embedding, and the original mould design was updated to include a sensor mounting channel and an antenna recess to accommodate the SAW sensor assembly. The stop-and-go method was used as previous authors have successfully exploited it for AM fabrication of smart components [19, 27, 28].

### SAW sensor

**Fig. 1:**
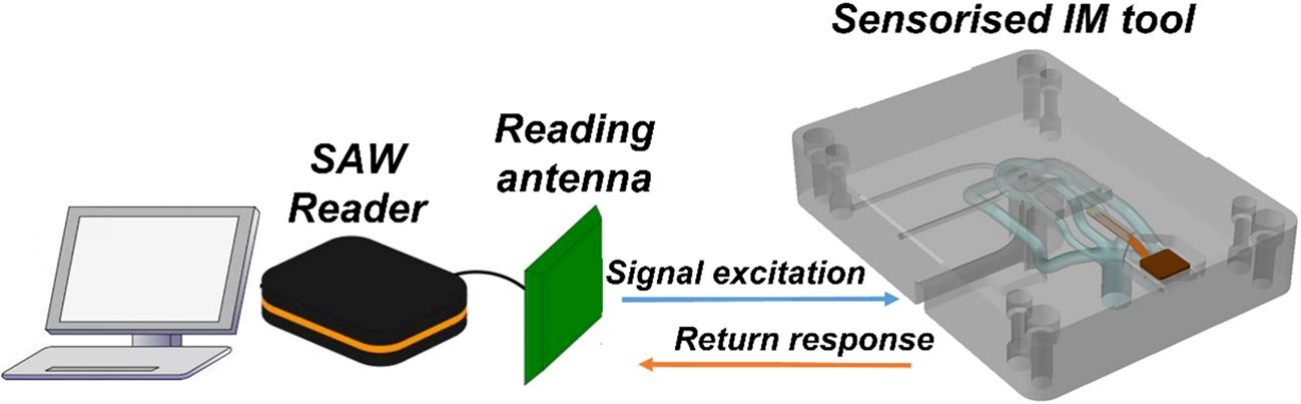
The schematic for IM experiments on industrial equipment using the IM tool with an embedded SAW sensor

The SNT2427BB2 model wireless temperature SAW sensor from SAW Components was selected for embedding into a smart, additively manufactured injection mould tool. The operating temperature of the SAW sensor is from − 40 to 200 °C, making it suitable for use in a range of IM applications and enabling use in the L-PBF metal AM stop-and-go approach. This is only possible through design considerations that isolate the sensor from the high temperatures experienced during fabrication. The sensor consists of the temperature sensor and transmission antenna, connected via a printed circuit board (PCB) ribbon cable. The cable contains an inductor to protect the sensor elements from electrostatic discharge. A frequency-modulated continuous wave interrogation unit, fabricated by SAW Components, was connected to the receiver antenna, and these were applied to register and record the temperature readings transmitted from the sensor antenna (Fig. 1).

### L-PBF process parameters

The L-PBF process was used to fabricate the smart injection mould tools in this study using an EOS M280 printer and gas-atomised stainless steel 316L powder provided by Carpenter Additive. While 316L stainless steel is uncommon for plastic IM applications, it is widely applied in the manufacture of polymer components for medical devices since it does not leach into the moulded component and demonstrates high oxidation resistance, attributes that make it the material of choice for production tools used in the industrial partner’s processes.

The IM tools were printed in an inert argon atmosphere using the following process parameters: laser power of 195 W, scanning speed of 100 mm/s, hatch spacing of 90 µm and layer thickness of 40 µm. The build plate temperature was maintained at 80 °C to minimise residual stresses and prevent warping of the parts. The scanning strategy used hatching with a rotation angle of 67° between the layers, and an overlap between laser passes of 0.12 mm was applied.

### Sensor embedding

**Fig. 2:**
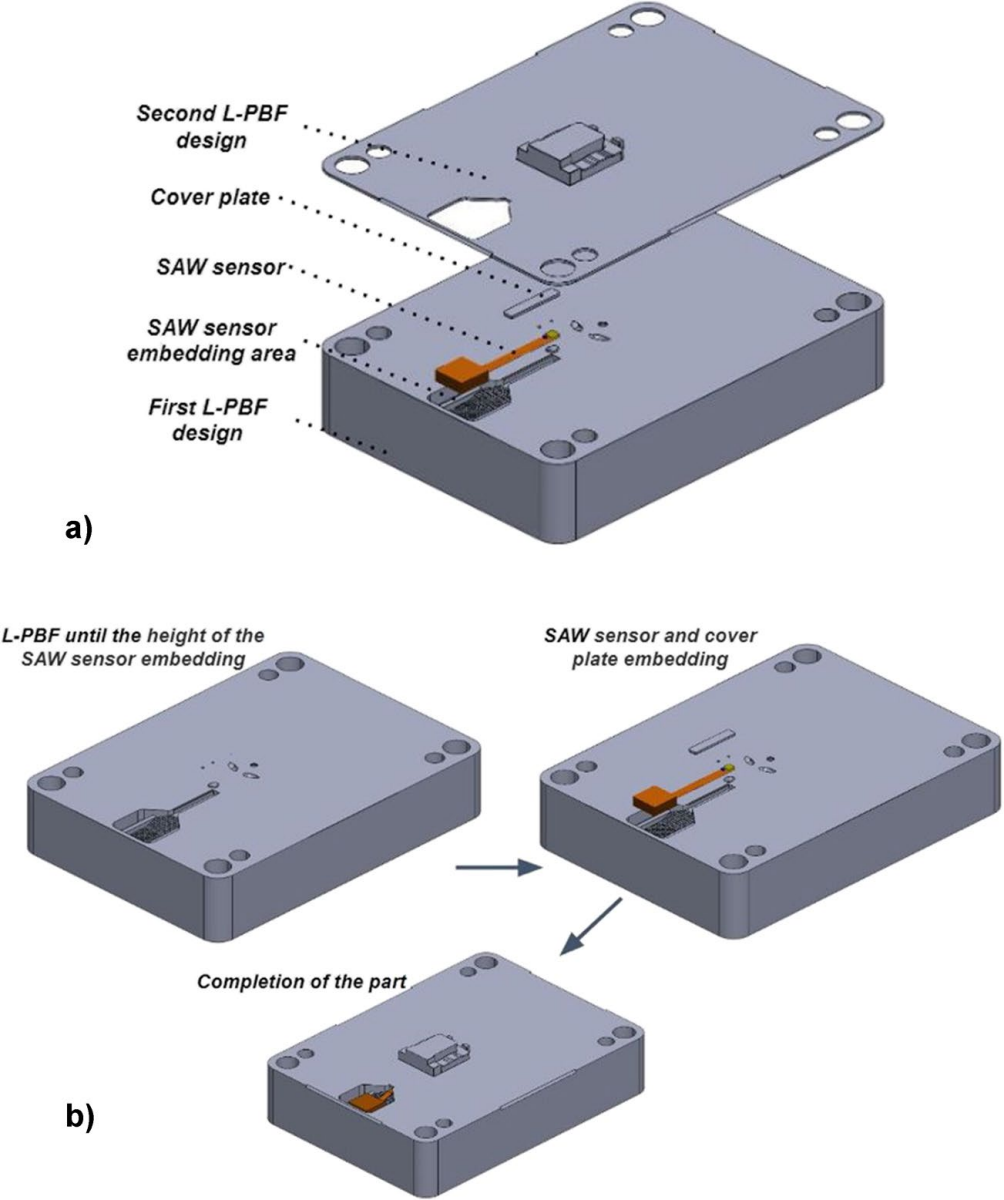
The CAD file of the fixed half core split into two sections (**a**) and scheme of the SAW sensor embedding using L-PBF stop-and-go strategy (**b**)

The successful stop-and-go strategy, as used by Tomaz et al. for embedding sensors into metallic parts, was applied to design the features necessary for embedding the SAW temperature sensor into the fixed half injection mould tool (core) (Fig. 2) [19]. The CAD file of the fixed half core was split into two sections, a bottom “base” and a top “cap”. Initially, the bottom part with a cut-out for the SAW sensor and antenna was printed. At this point, the printing process was paused, enabling the sensor and cover plate embedding as outlined in Fig. 2.

**Fig. 3:**
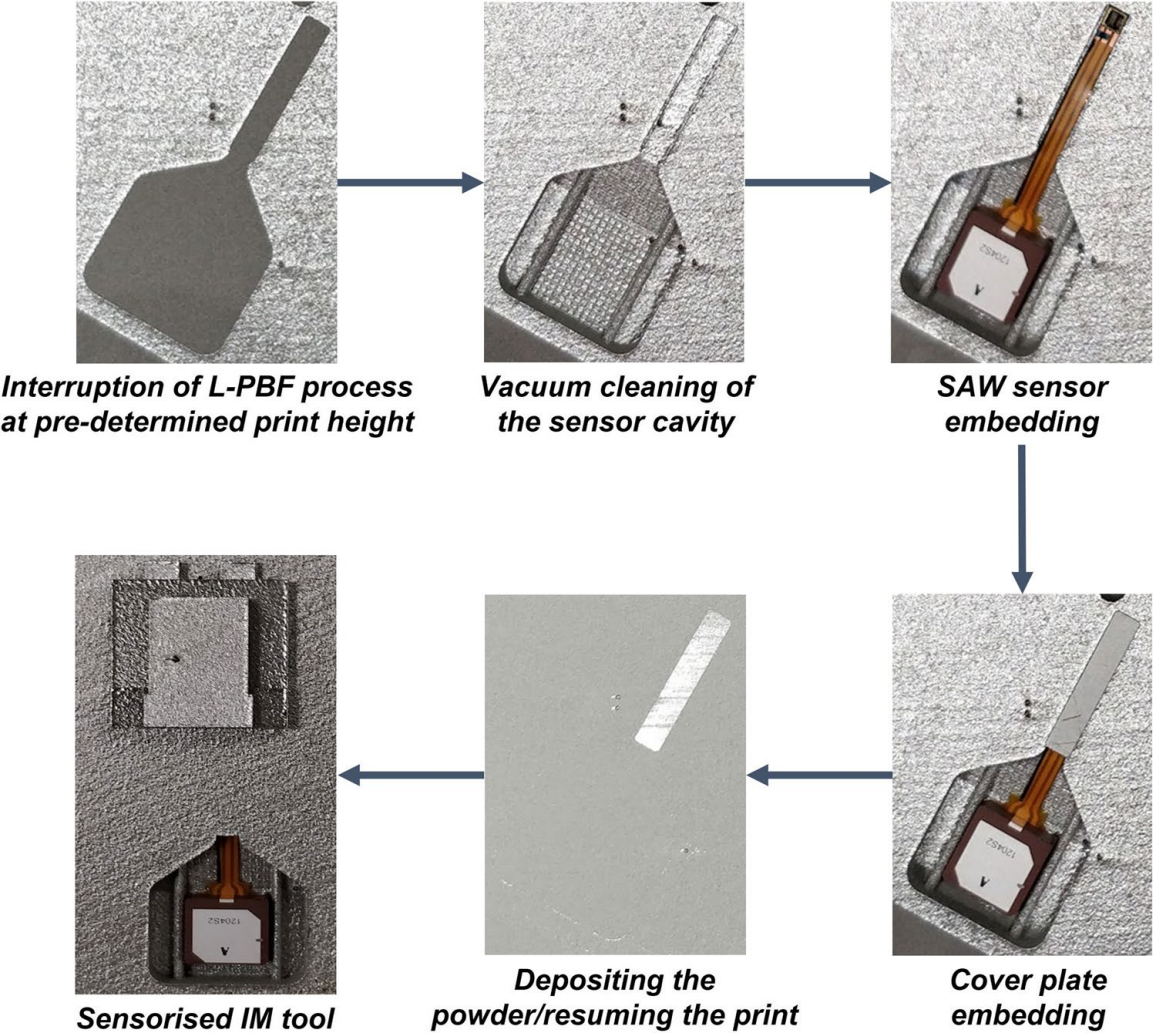
Scheme of the SAW sensor embedding

The pause was automated in the EOS machine software. A cover plate was designed to fit the sensor cable channel opening, with − 0.175 mm of clearance on either side to allow a precise fit; it was cut from a 0.5-mm-thick metal sheet using wire electric discharge machining (wire EDM). This thickness demonstrates the optimal sensor protection from the generated heat [19, 27]. The cover plate serves a dual purpose: as heat protection for the sensor and as a base for the first layer when the printing process is resumed. The build chamber was opened, and the cut-out for the sensor and cover plate was de-powdered using a wet separator before installing the sensor with the cover plate. The SAW sensor and cover plate were placed into their predefined locations. The cover plate was set in contact with the sensor, and the plate geometry was designed with a transition fit that acted as a clamp to prevent the sensor from moving during powder compaction. Tomaz et al. experimentally confirmed that a cover plate of 0.5-mm thickness is sufficient to protect the SAW sensor against the heat generated during the L-PBF process without exceeding the 200 °C upper operating limit of the sensor at the active region and ensuring uninterrupted sensor operation after the embedding [19] In addition, another study modelled and experimentally demonstrated that a plate thickness of 0.5 mm is sufficient to protect the embedded sensor during the metal AM process [27]. Finally, the part was recoated with powder, and the printing process resumed (Fig. 3).

The SAW sensor was embedded within 1-mm proximity of the outlet cooling channel, thus enabling it to acquire the temperature information of the cooling system. A number of additional factors, such as the L-PBF process constraints and predefined geometry of the injection mould tool, such as conformal cooling channels and ejector blades, determined the exact sensor position. The geometry of the sensor assembly required installation space to enable correct function and low-risk installation.

The L-PBF process uses a soft blade recoater to spread the powder layers. Therefore, after sensor embedding, all parts of the sensor and plate must be positioned below the level of the recoater trajectory to avoid a collision when the build process is resumed. In particular, because the height of the antenna is greater than that of the sensor and cover plate together, the cut-out for the antenna had to be designed to locate it below the recoater trajectory.

**Fig. 4:**
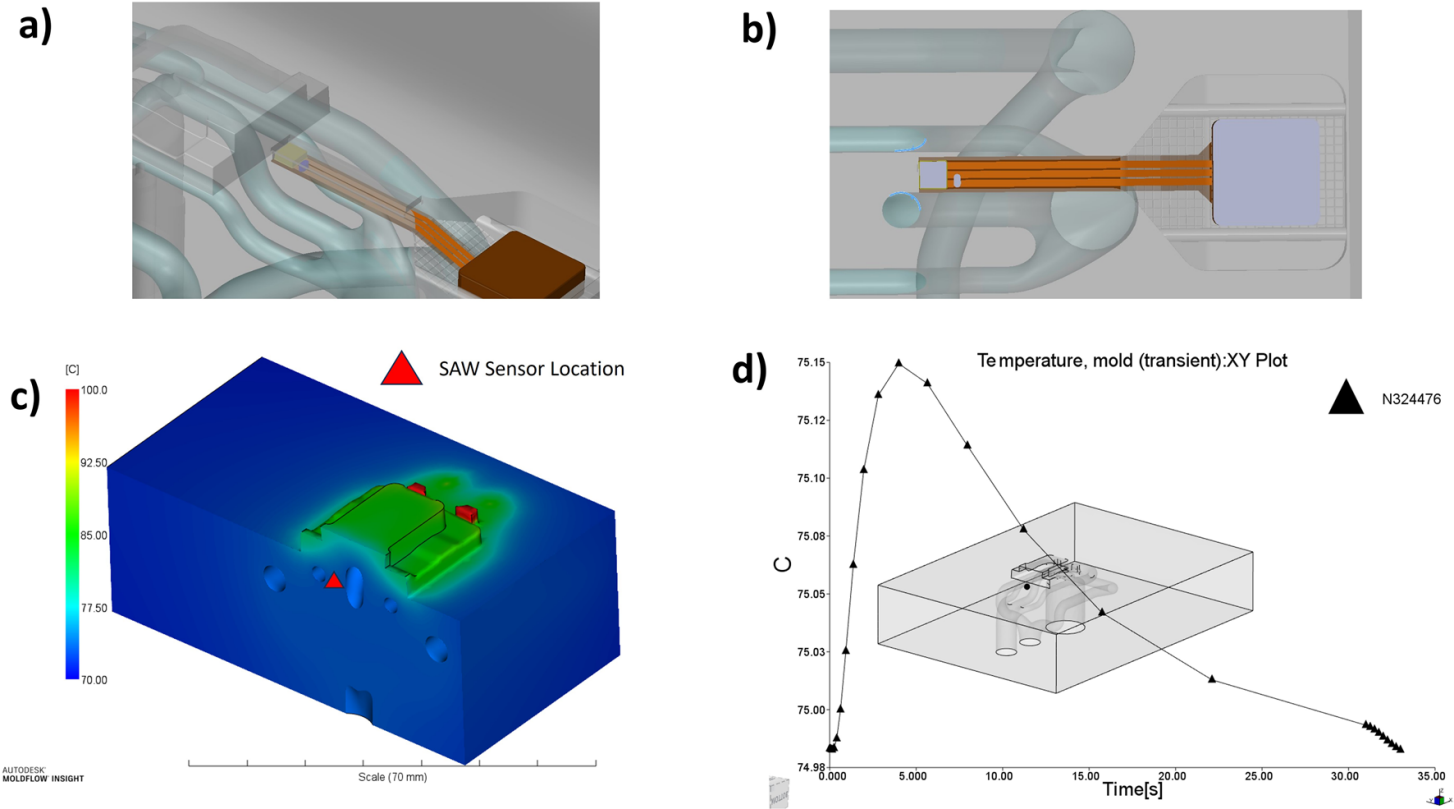
The proximity of the sensor to the conformal cooling channels (**a**, **b**) temperature contour plot of the cross-section at the end of mould filling (**c**) and transient temperature at the sensor location as a function of time from start of fill to end of cooling (*T*_melt_ = 195 °C and *T*_mould_ = 90 °C) (**d**)

The proximity of the sensor to the conformal cooling channels and the clamping surface (Fig. 4a, b) of the mould is another critical criterion. Due to space restrictions in the mould tool, only one location had sufficient space to mount the sensor assembly to obtain acceptable temperature readings from the cooling channels while addressing the other design constraints. To reduce the risk of thin-walled features failing under the cooling channel coolant pressure and the clamping pressure, it was chosen to maintain a minimum 1-mm wall thickness between the sensor, cooling channels and the mould clamping surface. This final position enables the sensor to obtain the temperature data during the process while minimising the risks of wall damage that could arise during the process.

Moldflow simulation was performed to determine the thermal profile (Fig. 4c) across the mould tool and transient temperature plot at the SAW sensor location throughout an IM cycle (Fig. 4d).

The risk imposed by continuing the print after the sensor was embedded had to be considered in the design. Extra space was designed between the antenna and melt pool for the remaining layers during the print. The maximum SLM process temperature near the protective layer can reach up to 926.85 °C [27]. Therefore, a 1.87-mm air gap between the sensor ribbon and the cover plate was designed to protect the circuit connections from laser-generated heat. The active region of the sensor was in contact with the plate for optimal temperature readings during the IM process.

The last constraint considered for the sensor embedding was sensor functionality. When printing the mould tool, completely enclosing the SAW sensor in 316L stainless steel would block signal communication between the sensor antenna and interrogation unit [16]. Therefore, an opening for the sensor antenna was incorporated into the tool design to enable uninhibited communication with the interrogation unit. In addition, sufficient clearance was ensured between the antenna and the opposite clamping surfaces of the mould tool to minimise the risk of thermal or mechanical damage to the sensor.

### Sensor protection during the post-processing

Typically, parts fabricated by L-PBF have high surface roughness and lower geometrical accuracy than CNC-machined parts. Consequently, post-processing is required to achieve the level of precision and surface finish needed, within the specifications for the moulding tools. The injection mould tools were therefore printed with 0.5-mm extra material on all critical faces.

After printing, the mould tools were separated from the build plate using wire EDM. In addition, the tooling underwent CNC milling, grinding and a number of mirror EDM processes to improve the surface quality and achieve tighter geometric tolerances. The general tolerance on the mould cavities was ± 0.025 mm with tighter tolerances for shut-off faces to prevent flashing, such as an H7/g6 transition fit tolerance applied to all hole- and shaft-based geometries. The required surface roughness for shut-off and moulding surfaces was 0.8 μm *Ra*.

**Fig. 5:**
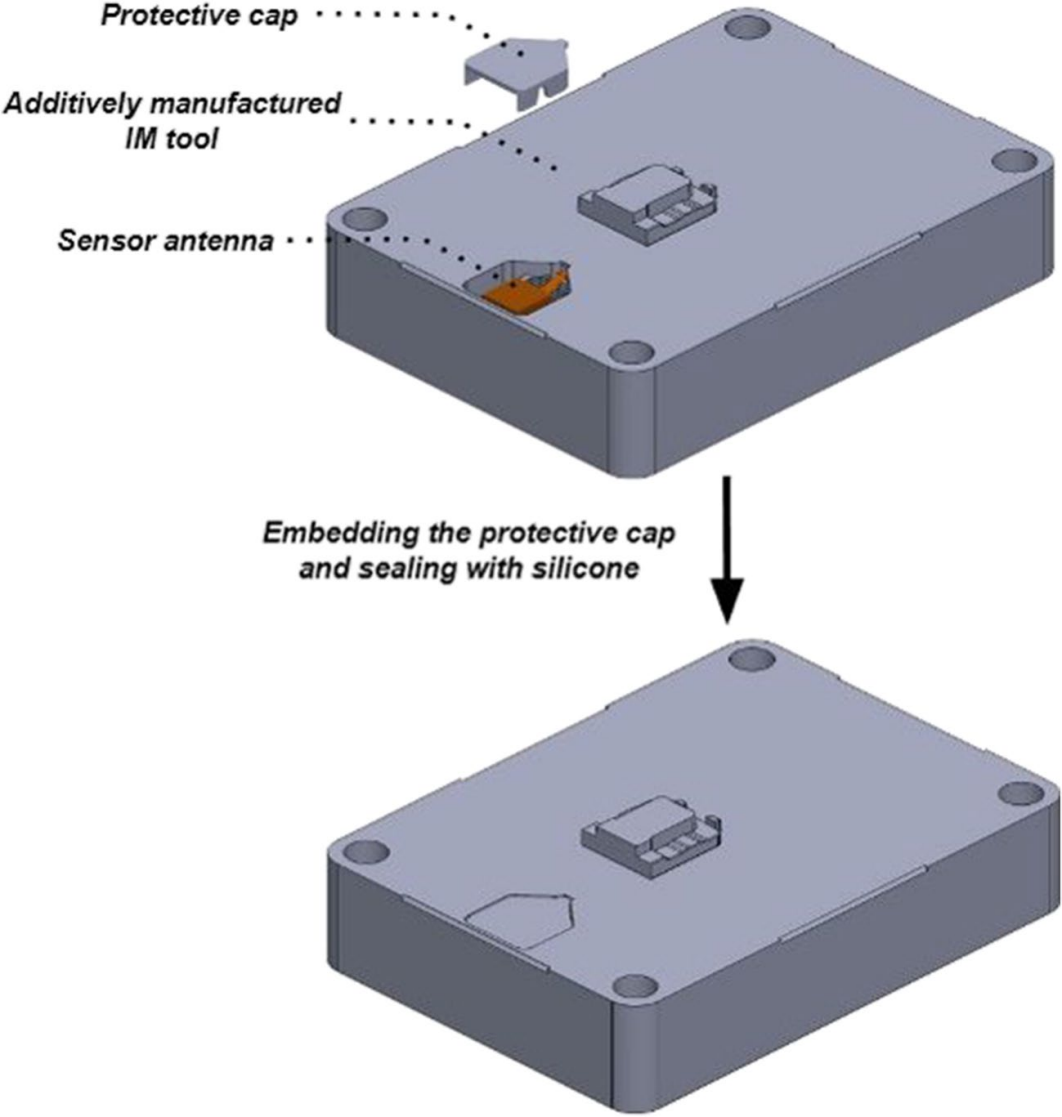
Schematic of the sensor protective sealing

During post-processing, sensor protection against cutting tools, swarf, dielectric fluids and coolants used in wire EDM and CNC machining is necessary. A protective cap was designed and additively manufactured from stainless steel 316L. The steel cap was positioned on top of the antenna, and silicone sealant was applied to create a watertight seal (Fig. 5).

### Vent plate embedding

**Fig. 6:**
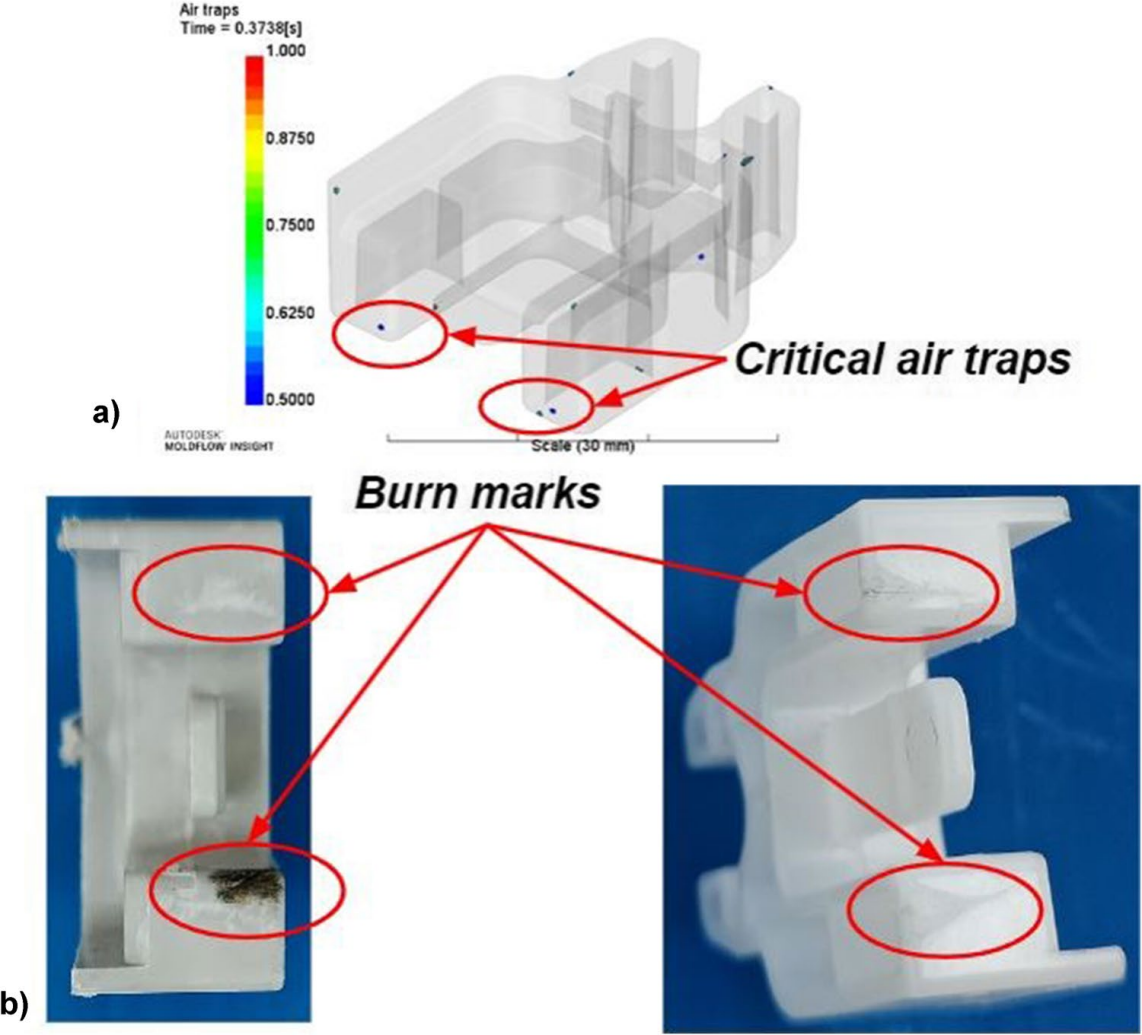
Moldflow simulation to investigate the location of air traps during the filling stage (**a**) and burn marks on polypropylene (left) and on POM Delrin® 500P NC010 (right) parts (**b**)

During the IM process, molten plastic is injected between two closed mould halves into a cavity which determines the shape of the fabricated part. The cavity becomes airtight once both halves are in contact under high clamping forces. Thus, it is necessary to evacuate the air which is inside of the mould. The compression of air slows the rate at which polymers fill the cavity and can lead to burn marks (Fig. 6b), since the highly compressed air combusts under high pressure; this is known as the diesel engine effect. Venting is, therefore, necessary to ensure that the molten plastic occupies the entire space within the cavity and that the air can escape during the filling stage.

A Moldflow simulation was conducted to investigate the location of air traps during the filling stage. Figure 6 a depicts the output from the air trap analysis; this was reviewed along with experimental outputs conducted in the same cavity design without any venting to assess where the burn marks occur. Figure 6 b shows the burn marks that occur when moulding with two different polymers. This collection of data confirmed the design location for the vents at the deepest part of the cavity furthest away from the gate. Other air traps identified in the simulation can dissipate through the clearance between ejector pins and the cavity, or through the shut-off faces on the cavities.

**Fig. 7:**
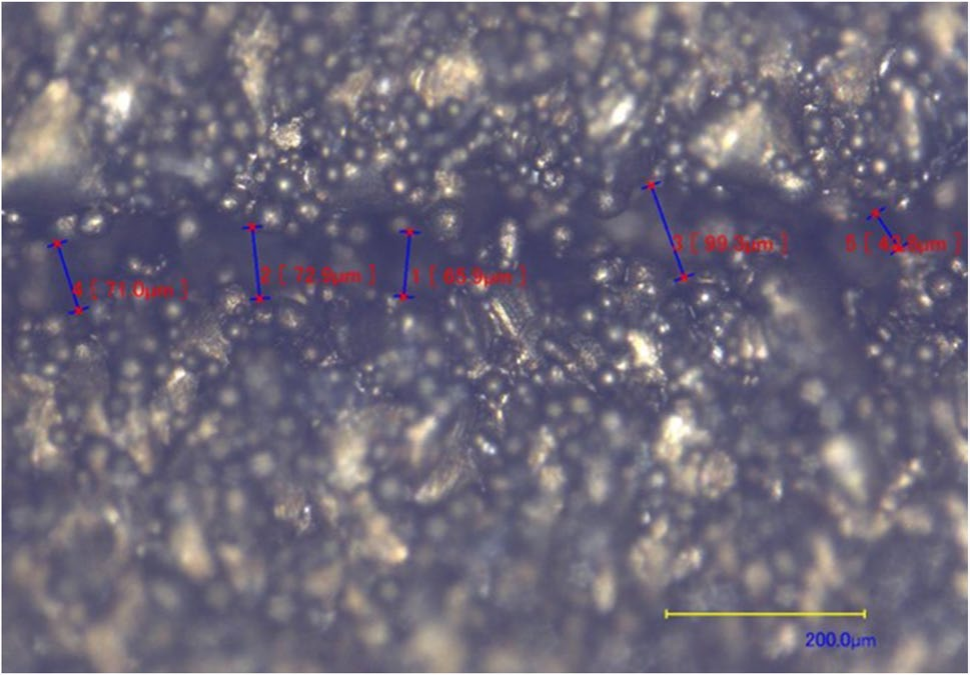
Microscope image of microchannel vent printed in vertical orientation

Vents are typically designed with a channel depth range of 0.01 to 0.05 mm and a channel width of 1.5 to 6 mm depending on the grade of polymer used [29]. Experiments were conducted to evaluate the capability of additively manufactured microchannels in both the vertical and horizontal orientations, using channel depths in the range of 0.01 to 0.05 mm. All of the horizontal experiments yielded no microchannel as the penetration depth of the melt pool is too great, causing the void to be filled. The trials on the vertical samples produced voids when printing the channels of 0.01-mm depth, but the surface roughness of the AM samples left semi-closed regions across the channel width (Fig. 7). Upon inspection of the vertical channels, it was identified that the risk of clogs was too high on all channel depths with rough channels, so an alternative approach was required.

**Fig. 8:**
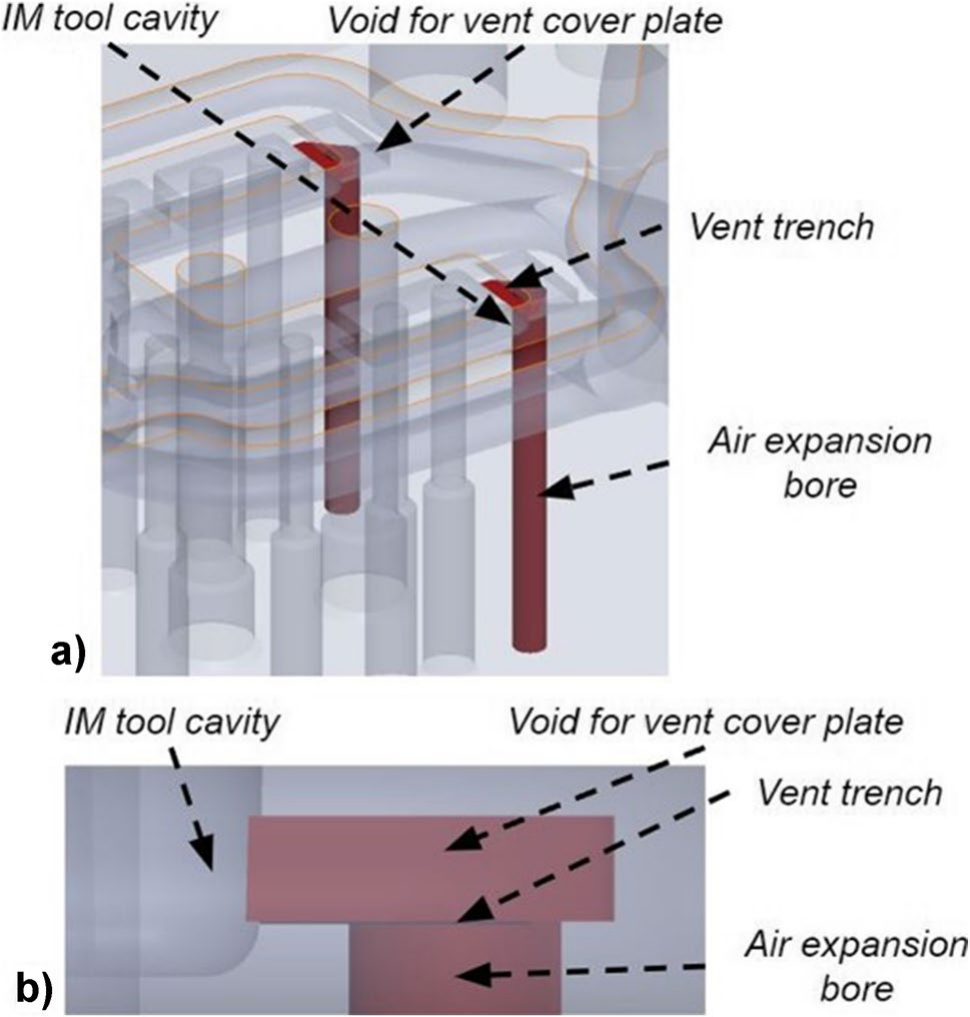
Air vent system design features (in red): isometric view of vent channels (**a**) and side profile view of vent design (**b**)

The vent design was reconsidered using the L-PBF stop-and-go method. The original mould tool design for the moving half tool was modified with two vent trenches, which connect the cavity to an air expansion bore (Fig. 8). The air vent trench of 0.02-mm depth was selected based on the manufacturer’s recommended vent design principles for the selected polymer (Delrin® 500P NC010). This gap is sufficient to evacuate the entrapped air without allowing the molten plastic to escape the mould tool. Additional space was designed above the 0.02-mm trench for embedding a 0.5-mm-thickness steel vent cover plate. The vent cover plates were cut to size using wire EDM and installed as a base for the first layer when resuming the printing process. The plate also serves to protect the vent channel from being closed by the melt pool. The use of this approach enabled the vent channel to maintain the integrity of the 0.02-mm-deep horizontal vent slot. Any overhang of these plates into the cavity space was cut during the post-processing of the cavity using a sink EDM process.

**Fig. 9:**
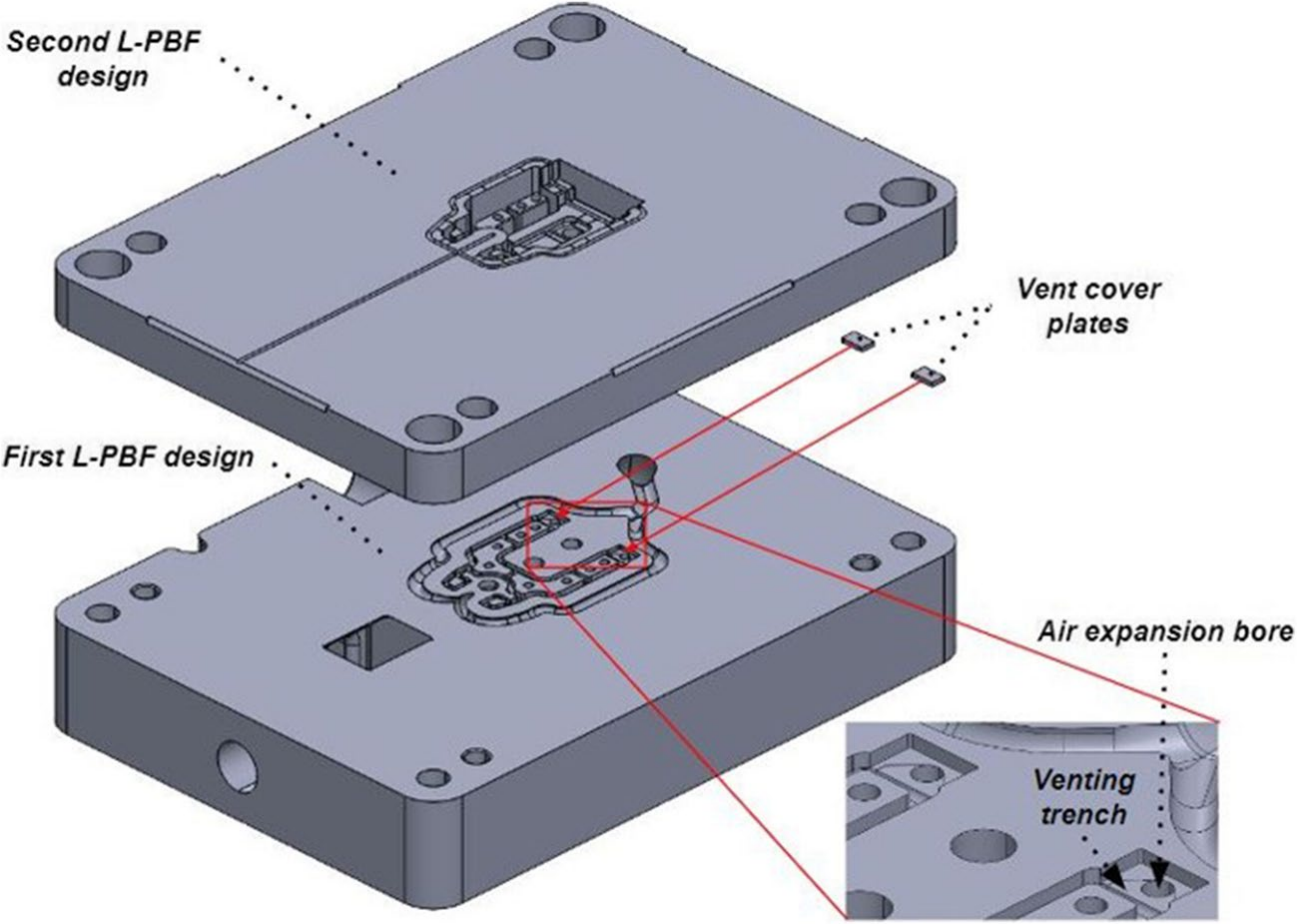
The scheme of the vent cover plates embedding using the L-PBF stop-and-go strategy

**Fig. 10:**
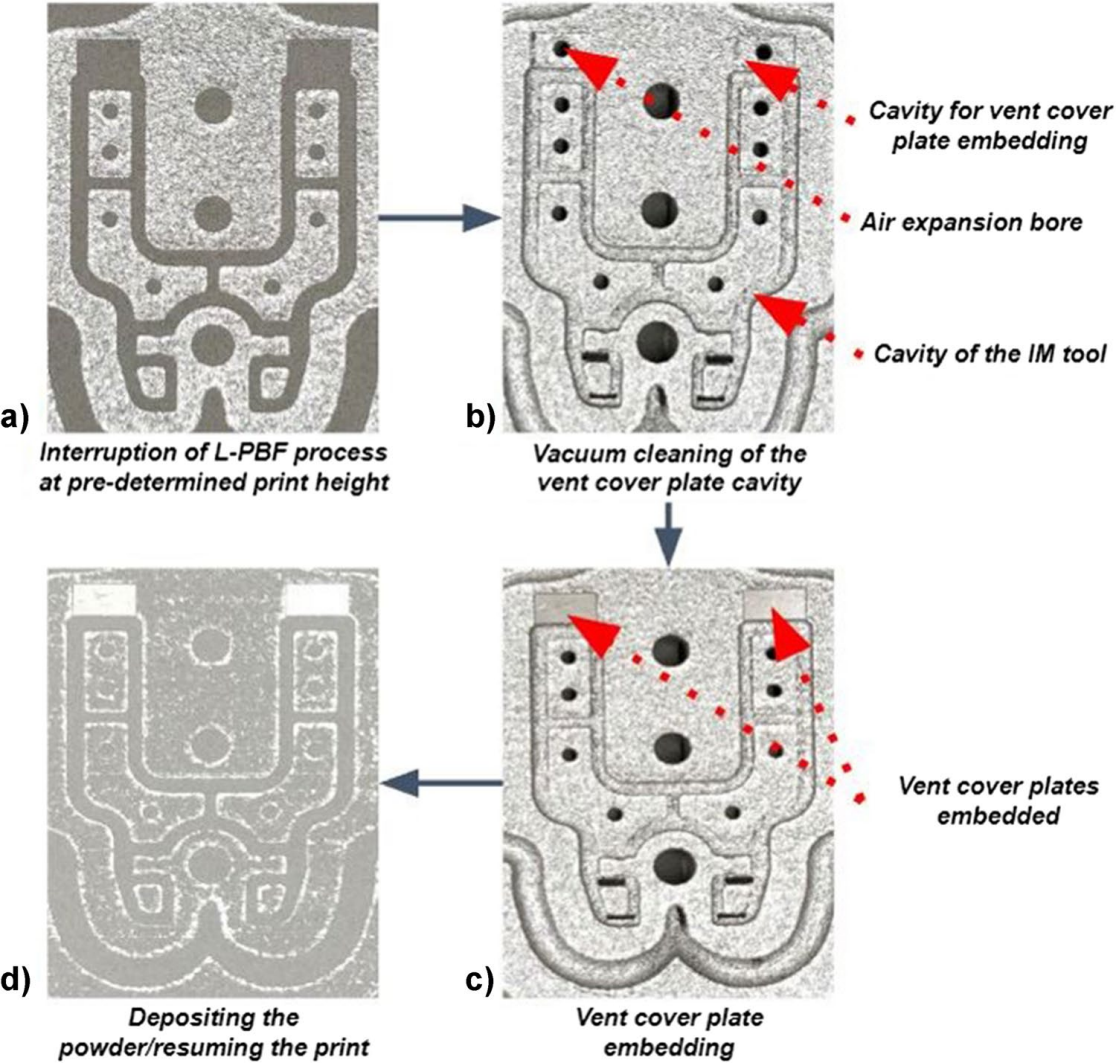
Scheme of embedding the vent cover plates: interruption of the printing process (**a**); de-powdering of the vent trench and vent cover plate cavities (**b**); embedding the vent cover plates (**c**); re-deposition of the powder and resuming the printing process (**d**)

Similar to the SAW sensor embedding procedure, for the L-PBF process, the CAD of the moving half tool was divided into a bottom and a top section (Fig. 9). The bottom part of the moving half tool was printed with the vent design features and space for the cover plate, and the printing process was then interrupted (Fig. 10 (a)). Next, the metal powder was vacuum cleaned (Fig. 10 (b)), and the vent cover plates were embedded (Fig. 10 (c)). Finally, the powder was re-deposited, and the printing process resumed (Fig. 10 (d)).

### Microstructure characterisation

The characterisation of the porosity and microstructure of the metal AM tool set was performed to determine the quality of the fabricated parts. Cross-sectional imaging, previously applied by other authors, was used in this study [19, 28]. The sample was initially cross-sectioned, embedded in epoxy resin and polished. Subsequently, the porosity was examined using a Keyence VHX 100R microscope and ImageJ software for pixel comparison. Finally, it was etched using Fry’s reagent (150 ml H_2_O, 50 ml HCl, 25 ml HNO_3_, 1 g CuCl_2_) for a melt pool analysis, which was performed using a Keyence VHX 100R microscope.

### Sensor testing in actual IM operations

The embedded SAW sensor performance and functionality within the smart tool set was analysed in an industrial IM process using two IM machines, a Fanuc Roboshot α-SiB 100-ton and Sandretto µ Micro 50 machines, respectively. The setup of the IM process using a sensorised injection mould tool is shown in Fig. 1.

**Fig. 11:**
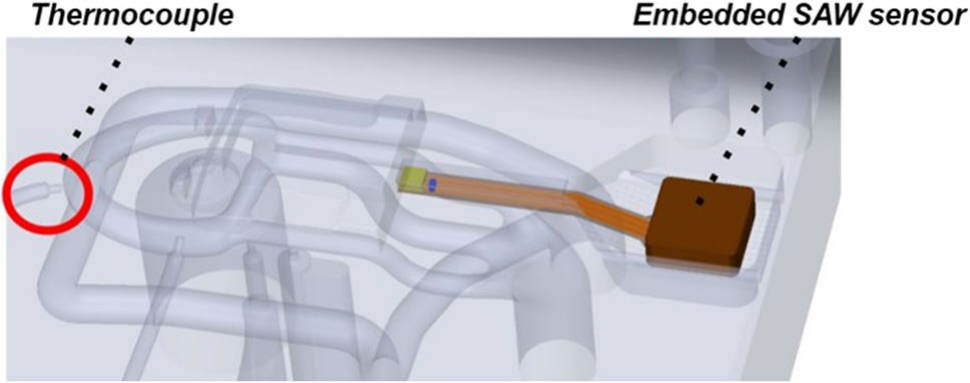
Schematic of the embedded SAW temperature sensor location and the calibrated thermocouple within the AM IM mould

The SAW sensor temperature calibration was performed according to the temperature measured by a calibrated thermocouple (type K IEC) located between two cooling channels within the same AM IM tool (Fig. 11). After the SAW sensor calibration, tests were performed to register temperature changes in the cooling channel outlet region during the IM cycle.

## Results and discussion

### Microstructure

**Fig. 12:**
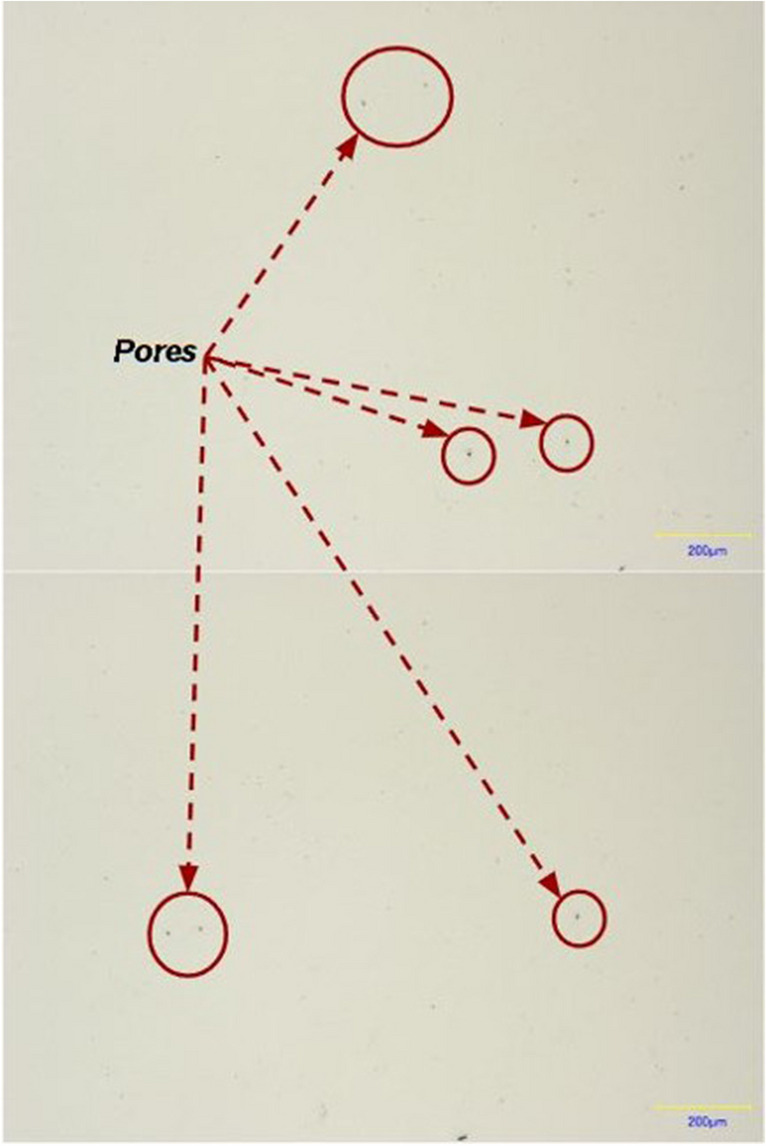
Porosity analysis

The porosity analysis (Fig. 12) demonstrated that the part density is 99.9%. The porosity comprises spherical pores with an average size of 10 μm; the pores are not interconnected. Neither additional cracks nor visible defects were detected during the analysis.

**Fig. 13:**
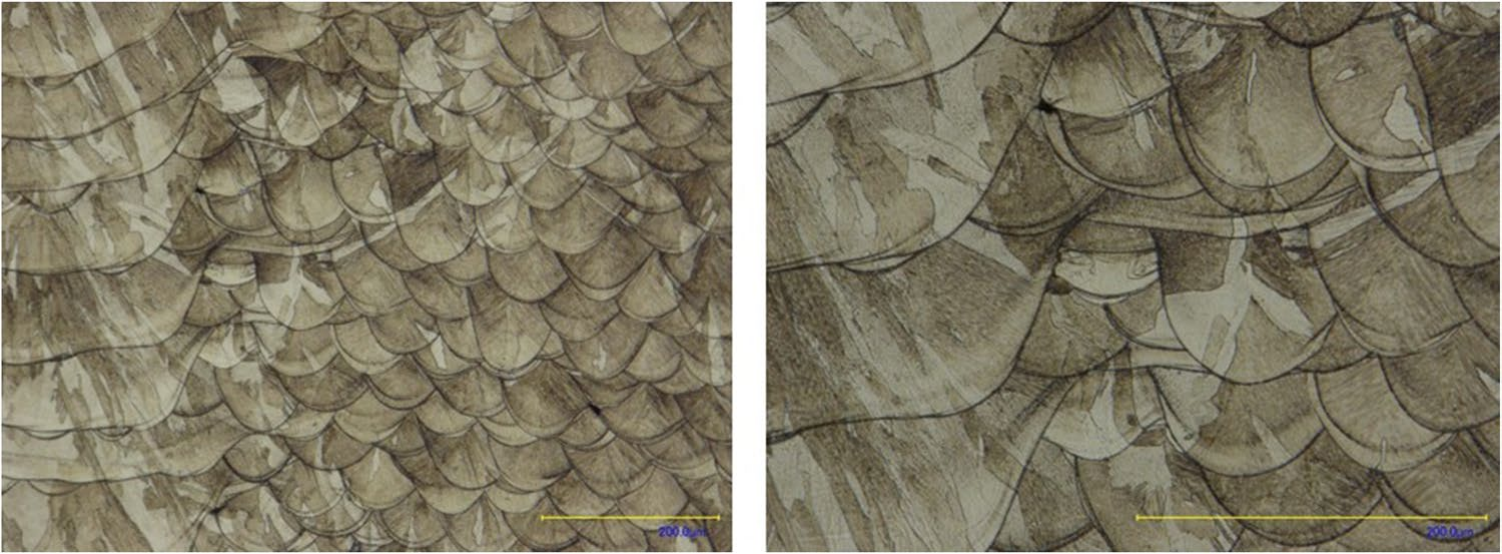
Microstructure analysis

The microstructure analysis (Fig. 13) demonstrates a bonding between the subsequent layers with no visible contamination detected. In addition, no visible defects are apparent between melt pool tracks.

### Sensorised injection mould tool

**Fig. 14:**
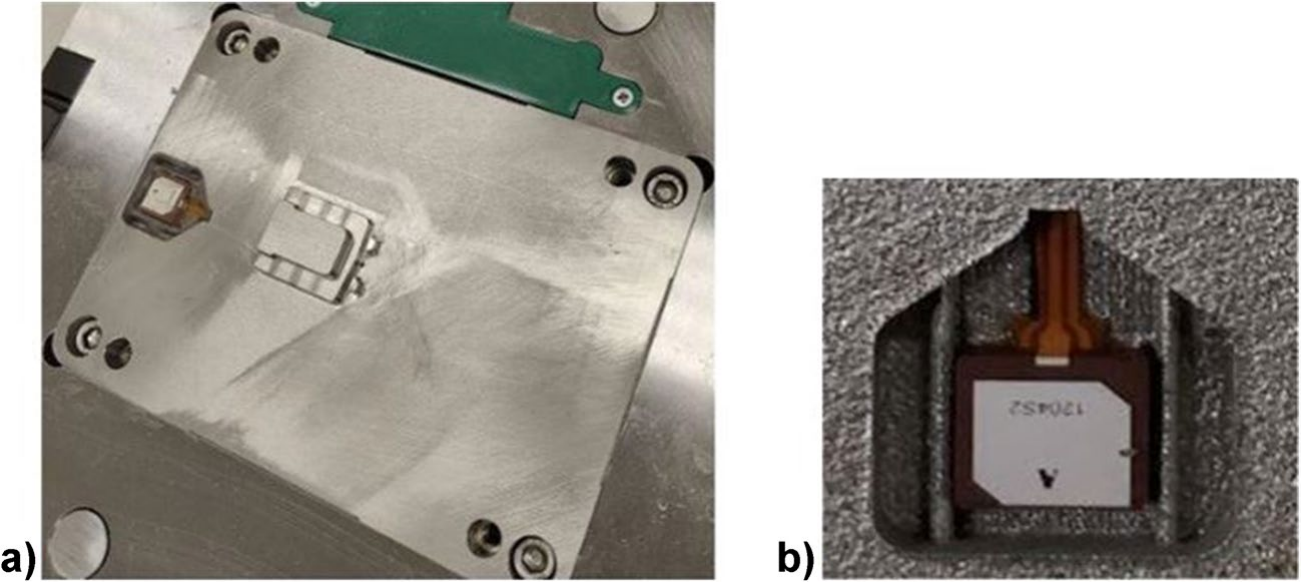
Sensorised injection mould tool installed in the mould base (**a**) and embedded SAW sensor (**b**)

The post-processed fixed half injection mould tool, when installed in the mould base, is represented in Fig. 14a. A visual inspection of the fixed half tool with the embedded SAW sensor (Fig. 14b) demonstrates no visible deformations or damages caused during the printing or post-processing. The injection mould tool was successfully installed into the mould base without additional geometry modifications.

### Venting results

**Fig. 15:**
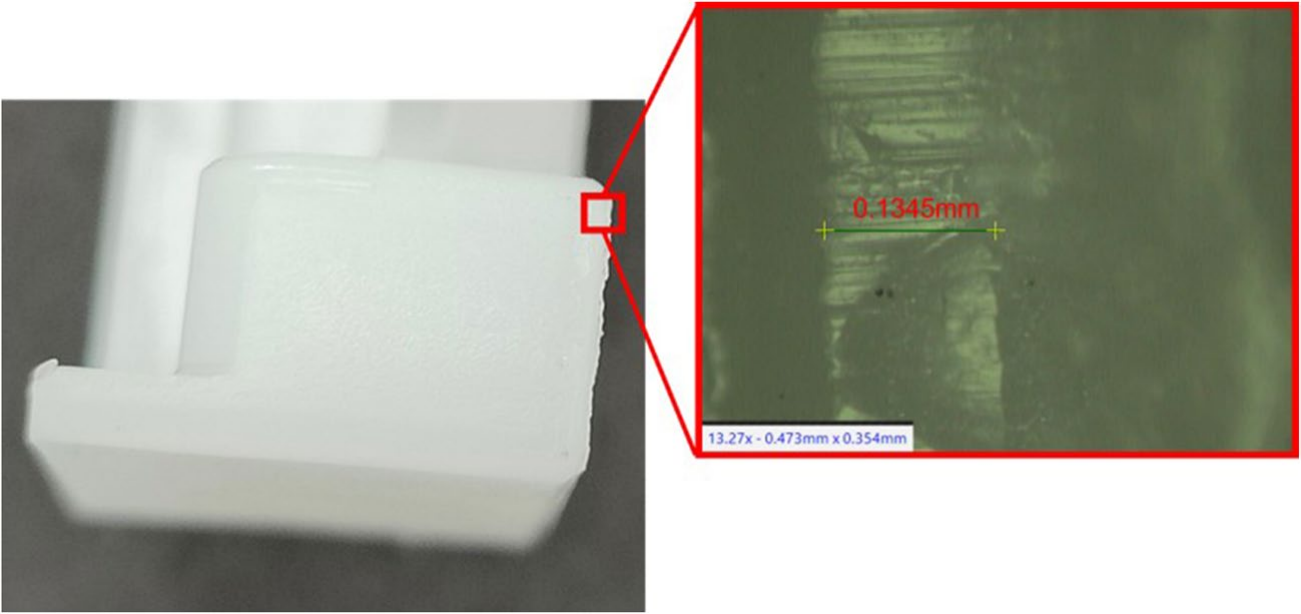
Vent witness mark on an injection moulded part from AM tools monitored by the SAW sensor with AM deep channel vents

The inspected injection moulded parts demonstrate no visible burn marks or other defects during any of the process trials (Fig. 15). The proposed concept design for the embedded venting system as described in this research enables excellent air evacuation, thus avoiding potential defects in the fabricated part and ensuring that the entire space within the cavity is filled with molten plastic. This can be seen in Fig. 15, where witness marks are visible in the location of the vents, as is the absence of burn marks. The witness mark indicates the channels were slightly larger than the designed 0.02-mm depth. This is likely caused by the rough surface of the sintered channel and possibly some un-sintered powder partially bonded to the channel surface, causing the plate not to sit flush on such a small area. In addition, the vent was implemented on the moving half of the IM tooling, which is opposite to the sensorised half; therefore, the presence of the vent void had no impact on the SAW sensor performance.

### SAW sensor testing

**Fig. 16:**
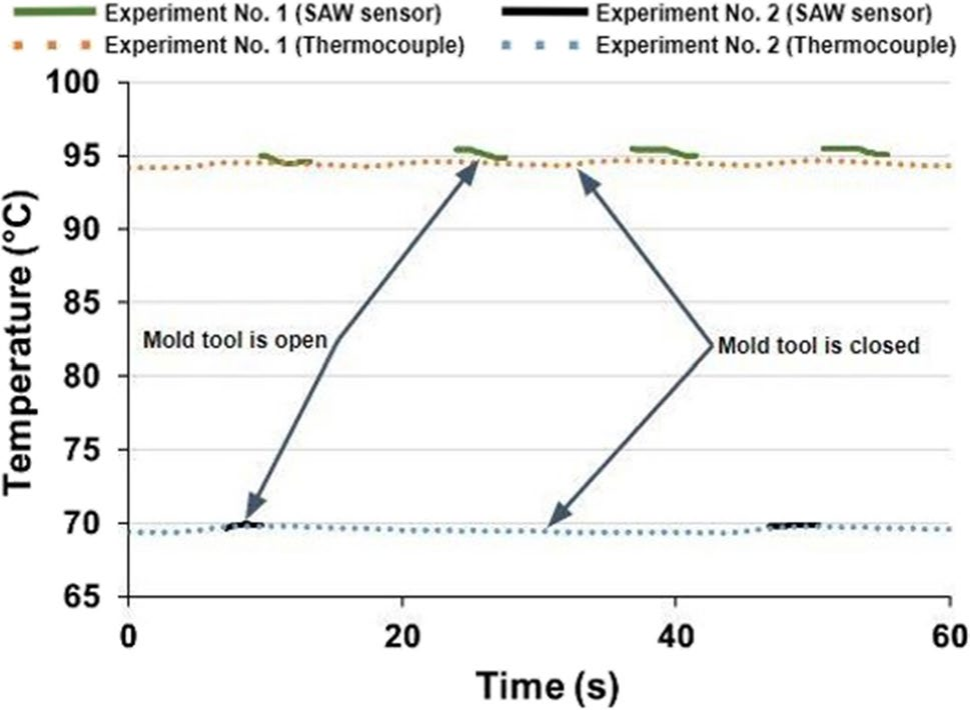
Temperature readings of IM obtained by the embedded SAW sensor and type K IEC thermocouple

Figure 16 represents temperature measurement results obtained using the SAW sensor during the IM on a Fanuc Roboshot α-SiB 100-ton machine. These results demonstrate the functionality of the sensor after the embedding process. The sensor is operating during the mould tool opening and ejection cycles; however, the mould tool closing stage of the IM cycle for injection, packing and cooling resulted in signal loss. Two experiments under different polymer and mould temperatures were performed.

**Fig. 17:**
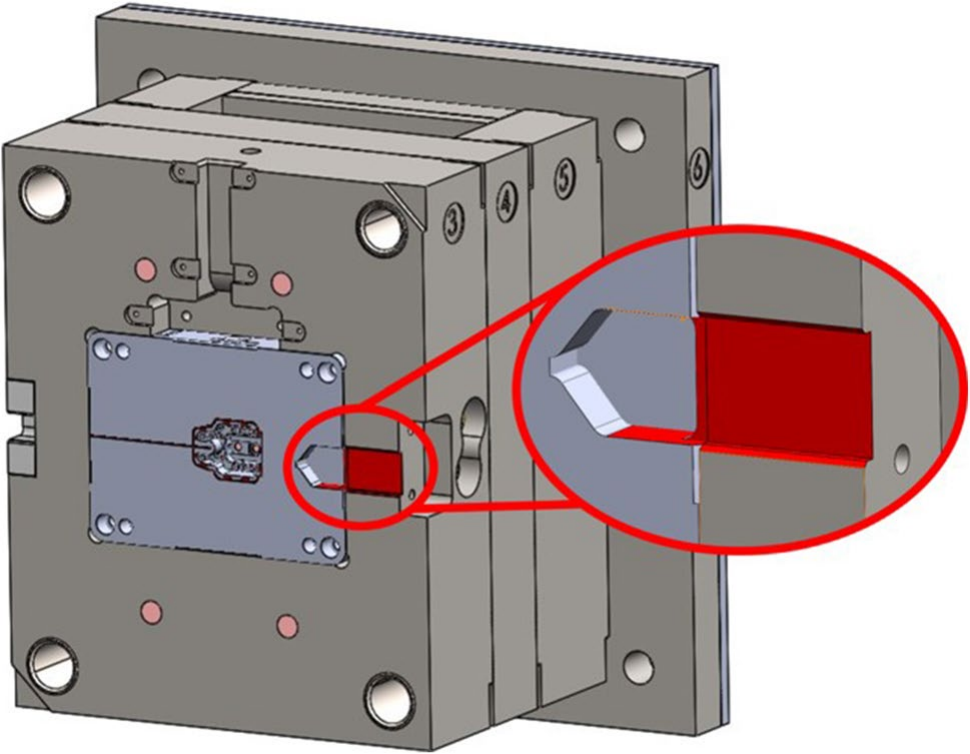
CAD model of the design modification on the moving half AM mould tool and mould base

The mould base and mould tool were modified by milling a 3-mm channel in the moving half of the injection mould tool and mould base (Fig. 17). This was proposed to facilitate the signal transmission while the mould base was closed.

**Fig. 18:**
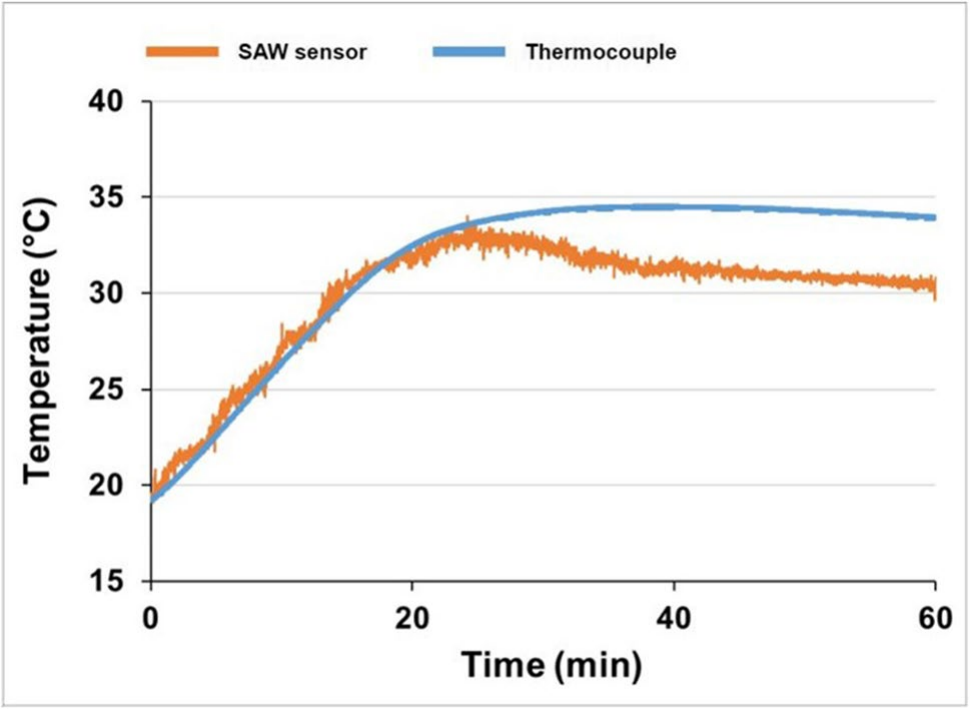
A temperature reading from the SAW sensor and type K IEC thermocouple in the closed IM mould base and AM tool

After the tool was modified, the mould base was re-assembled and benchtop testing was performed to evaluate signal reading capability with a closed mould tool. To do this, the mould tool was closed, and an external heat source was applied to increase the mould base temperature gradually. The temperature reading of the SAW sensor inside the closed tool is represented in Fig. 18.

The continuous temperature curve obtained during the benchtop testing demonstrates the functionality of the SAW sensor in a closed injection mould tool after the design modification. Therefore, the SAW sensor testing in IM process conditions was repeated. While testing the modified mould base on a Sandretto µ Micro 50 machine, the signal was interrupted when the mould was closed. The main reason for that was that the cooling inlet and outlet pipes from the temperature controller were coming into contact with the antenna when the mould tool was closing, thus causing the antenna to move and disrupt the continuity of the signal.

**Fig. 19:**
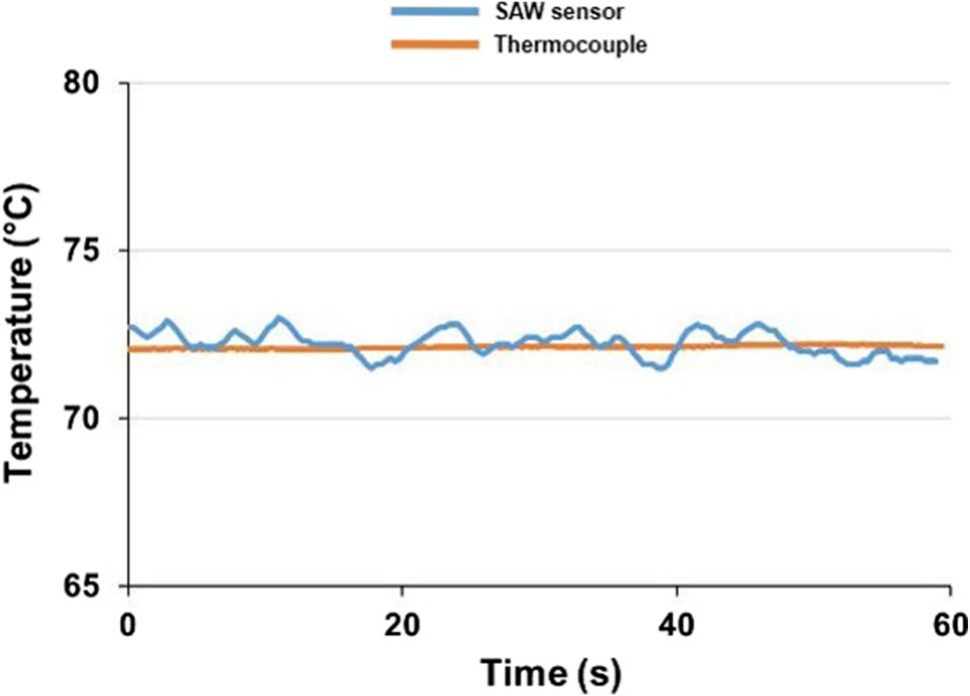
Temperature readings of the IM process obtained from the embedded SAW temperature sensor and the type K IEC thermocouple after removal of cooling pipes

The location of the embedded SAW sensor was defined during the design and fabrication; this consequently constrained the location of the signal transmission channel; i.e. the channel was constrained to the same side of the mould base as the cooling pipes. Ideally, the machined signal transmission channel should be located on the opposite side of the coolant pipework, as this would avoid any contact between the cooling pipes and the antenna receiver. Therefore, to achieve a signal for the full duration of the moulding cycle, it was decided to remove the cooling pipes for the purposes of testing and the tests were repeated on the sensorised tool (Fig. 19).

Figure 19 demonstrates that when there are no physical interactions with the receiver antenna caused by cooling pipes, it is possible to achieve continuous temperature readings throughout the IM cycle. Figure 19 verifies the simulated results (Fig. 4c, d) and demonstrates that the temperature fluctuations are within 1 °C, since it is near the inlet of the cooling channel.

The SAW sensor applied in this research demonstrated higher sensitivity to temperature changes in comparison to the type K IEC thermocouple. However, higher signal variation in comparison to that of the in-mould thermocouple was detected after closing the IM tool, which suggests that further work on the improvement of signal transmission and noise reduction of the in-mould SAW sensor is required, as embedding such devices into metallic components is not widely applied.

Therefore, it is particularly important to continue this study by focusing future research on the waveguide design and implementation, as this would result in significant signal improvement of the SAW sensors embedded into metallic components. Finally, wireless temperature sensors such as the SAW sensor applied in this study are traditionally a challenge to embed into metallic components, yet bring value to the users by providing information rich process data, without requiring any wiring which can often make it impractical to locate the sensors in similar locations.

## Conclusions

The wireless temperature SAW sensor was successfully embedded into an additively manufactured, stainless steel 316L injection mould tool applying the L-PBF stop-and-go approach. The fabricated injection mould tool demonstrates no visible defects with a part density of 99.9%. The obtained results demonstrate that the sensor maintained its complete functionality after the embedding and post-processing stages and functions with temperature data acquisition throughout the IM process. This proves the embedding method and the use of the wireless SAW temperature sensor to make smart AM IM tools with real-time process monitoring and control abilities. Some minor design challenges were overcome to ensure the proper operation of the wireless sensor during the full IM cycles on industrial IM machines.

In addition to sensor embedding, this study also addressed the concept of a novel print-in-place vent design, which was successfully implemented in an area of the mould cavity that is inaccessible to conventional machining techniques. This enabled the IM of parts without burn marks, whereas cavities experimentally tested without venting resulted in parts with burn marks in the same locations as was predicted through air trap simulations.

Significant future developments in the sensor technologies are anticipated; such developments may include miniaturised sensors with the ability to store data locally. Such technology will enable the minor design challenges in this study to be overcome. However, this paper introduces the design flexibility offered by embedding wireless sensors into additively manufactured injection mould tools.

Nevertheless, during the study, several suggestions for future research were formulated. Ideally, the SAW sensor should be located on the opposite side of the cooling channel inlet and outlet pipework, as this would avoid the contact between cooling pipes and antenna receiver, i.e. signal disruptions. Alternatively, an antenna receiver of a smaller size could be considered, as this would enable greater flexibility in the sensor location. Alternative venting strategies and designs were formulated but not tested such as using AM porous vent surfaces in the AM cavity as opposed to specific targeted venting that is based on conventional vent designs. To the best of the authors’ knowledge, this is the first known application to develop and implement embedding strategies for a wireless temperature SAW sensor and novel print-in-place vents in functional metal IM tools, which highlight the future benefits of AM for the IM industry.
